# Global Research Hotspots in Venous Thromboembolism Anticoagulation: A Knowledge-Map Analysis from 2012 to 2021

**DOI:** 10.1155/2023/4717271

**Published:** 2023-11-17

**Authors:** Jia Wang, Yang-Xi Liu, Yi-Dan Yan, Li Liu, Chi Zhang, Mang-Mang Pan, Hou-Wen Lin, Zhi-Chun Gu

**Affiliations:** Department of Pharmacy, Renji Hospital, Shanghai Jiao Tong University School of Medicine, Shanghai 200127, China

## Abstract

**Background:**

Venous thromboembolism (VTE) is a common cardiovascular disease that seriously threatens human lives. Anticoagulant therapy is considered to be the cornerstone of VTE treatment. An increasing number of studies has been updated in the VTE anticoagulation field. However, no bibliometric analyses have assessed these publications comprehensively. Therefore, our study aimed to analyze the global status, hotspots, and trends of anticoagulant therapy for VTE.

**Methods:**

The relevant literature on VTE anticoagulation published between 2012 and 2021 was retrieved and collected from the Web of Science Core Collection database. VOSviewer, Cooccurrence Matrix Builder, gCLUTO, and some online visualization tools were adopted for bibliometric analysis.

**Results:**

A total of 15,152 related articles were retrieved. In recent years, the research output of VTE anticoagulation gradually increased. The United States was the most productive country. International cooperation is concentrated in North America and Europe; the most influential documents, journals, authors, and organizations were also from these two continents. Research hotspots mainly focus on clinical guidelines, VTE in special populations, non-vitamin K oral anticoagulants (NOACs), and parenteral anticoagulation. The research frontiers and trends include the assessment of NOACs and the antithrombotic management of VTE complicated with coronavirus disease 2019 (COVID-19).

**Conclusion:**

This bibliometric analysis provides a systematic overview of the VTE anticoagulation research, which will facilitate researchers to better understand the situation of VTE anticoagulation. Future studies should be dedicated to NOACs application and VTE-combined COVID-19 patients.

## 1. Introduction

Venous thromboembolism (VTE), comprising deep vein thrombosis (DVT) and pulmonary embolism (PE), is the third most frequent cardiovascular complication in hospitalized patients [1, 2]. Although the exact incidence of VTE is unreported, an estimated incidence of first VTE is 0.7 to 1.4 per 1000 person-years [3, 4]. Meanwhile, VTE can lead to severe complications, including postthrombotic syndrome for DVT, chronic thromboembolic pulmonary hypertension for PE, and death [5]. VTE is also characterized by high hospitalization costs, with estimated annual costs ranging from $13.5 to $27.2 billion in the United States [6]. Anticoagulant therapy is the standard treatment for VTE, consistently recommended by various guidelines [7–9]. However, the anticoagulant recommendations in different VTE settings are diverse, especially in patients with cancer, orthopedic surgery, antiphospholipid syndrome (APS), pregnant women, and children [10–14]. Following the rapid advances in understanding VTE and broadening approved indications of nonvitamin K oral anticoagulants (NOACs), it is necessary to summarize the progress, hotspot, and trend of VTE anticoagulation research.

Bibliometrics, which has rapidly developed in medical research, is widely used to quantitatively analyze knowledge structures and research trends in a specific area [15, 16]. The Web of Science Core Collection (WoSCC) database is a platform that contains representative and pioneering scientific research articles, which was the preferred retrieving source for bibliometric analysis in the medical field [17, 18]. The VOSviewer is a software tool to build bibliometrics visualizing network maps based on the search outcome of the WoSCC database. In addition, biclustering analysis can efficiently discover global and local information by simultaneously clustering the row and column data matrix, which is a novel bibliometric methodology to explore medical research hotspots [19].

There has been no bibliometric analysis conducted regarding VTE anticoagulation until now. Most of the literature on VTE anticoagulation research is limited to reviews that list or summarize the existing research and lack analysis of research hotspots or development trends, failing to provide substantive suggestions to scholars [11, 20]. The present study is the first to conduct literature bibliometric and visual analysis involving VTE anticoagulation. Compared with previous review articles, this study provides the characteristics, internal relationships, and scientific structure of VTE anticoagulation research based on the contents of authors, journals, institutions, countries, and keywords. More importantly, biclustering analysis is carried out to provide future trends for researchers and clinicians in this field.

## 2. Methods

### 2.1. Search Strategy and Data Collection

Web of Science (WoS, Clarivate Analytics, Philadelphia, PA, USA), which contains more than 12,000 international academic journals, is one of the most comprehensive and authoritative database platforms for obtaining global academic information. Moreover, apart from the general literature search, it also possesses an essential function of citation index searching, which helps assess the academic performance of literature in a specific field [21, 22]. Literature on VTE anticoagulation was retrieved from the WoSCC Science Citation Index Expanded (SCI-EXPANDED) database, published from January 1, 2012, to December 31, 2021. The search terms were designed according to previous studies and listed in Table S1 [23, 24]. Document type was limited to the article, and the language was restricted to English. The complete information of each included article, containing the title, authors, year of publication, affiliation, abstract, keywords, language, and citations, was exported into a text format. All literature was searched and downloaded independently by two reviewers (J.W. and Y.L.). Also, disagreements were discussed with the senior author (Z.G.) to reach a consensus.

### 2.2. Bibliometric Analysis

The essential characteristics of the included articles, containing the total number of literature, the ranking of affiliated institutions, productive funding agencies, research fields, and authors, were analyzed using intrinsic functions of the WoSCC database and Microsoft Excel (2019). Furthermore, annual publication trends were analyzed through the Online Analysis Platform (https://bibliometric.com/) [25]. VOSviewer (version 1.6.10), created by Leiden University, Leiden, the Netherlands, was applied to generate different scientific landscapes and networks. The construction of networks was mainly based on coauthorship, cooccurrence, citation, bibliographic coupling, or cocitation and themes. Coauthorship relations are confirmed when the authors, institutions, and countries or regions were cosigned in published papers; co-occurrence analysis calculates correlation strength by the frequency of occurrences at the same time between keywords. The citation analysis refers to the connection between the two documents by direct reference. Bibliographic coupling analysis assesses the similarity between two pieces of literature by examining the references they share, and cocitation analysis evaluates the relationship between two pieces of literature by analyzing how often they are cited together. In the overlay visualization map, it is worth noting the node colors represent the average publication year. Bibliographic Item Cooccurrence Matrix Builder Version 2.0 (BICOMB 2.0), designed by Professor Lei Cui from China Medical University, was used to retrieve a high-frequency keywords-source articles matrix [23]. Then, the matrix was input into gCLUTO 1.0 (Graphical clustering toolkit, gCLUTO; Karypis Lab, 2014) software to achieve mountain and matrix visualizations. The parameter settings of gCLUTO are adjusted to the suitable for biclustering analysis [26]. Finally, the repeated bisection was selected as the clustering method, the similarity function chose cosine, and *I*^2^ was set to the criterion function.

## 3. Results

### 3.1. Publication Output Patterns

**Figure 1:**
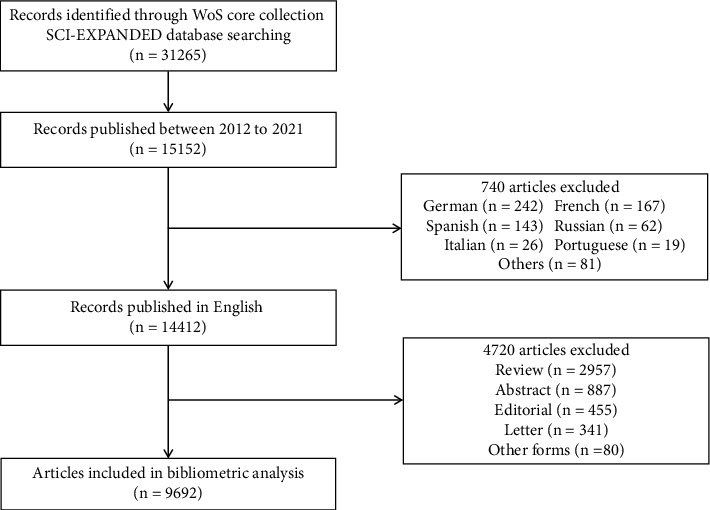
Flowchart for retrieved publications. WoS: web of science; SCI-EXPANDED: science citation index expanded.

Totally, 15152 articles involved in VTE anticoagulation topics were obtained in the WoSCC database from 2012 to 2021. There were 740 articles excluded due to language restrictions. Another 4720 articles, containing 2957 reviews, 887 abstracts, 455 editorials, 341 letters, and 80 other publication forms, were eliminated because they were nontarget article forms. We finally included 9692 articles that met the eligibility criteria for further bibliometric analysis (Figure 1). 47.57% (4610) of the publications were open access.

**Figure 2:**
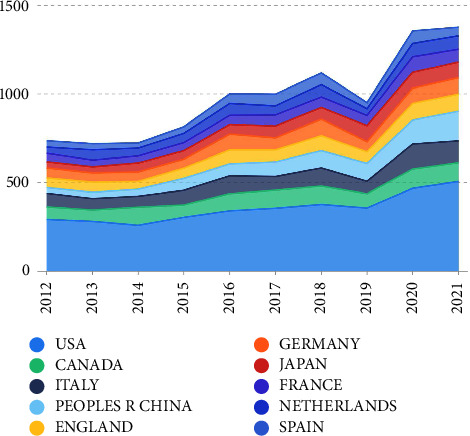
The annual growth trends of the top 10 productive countries in the VTE anticoagulation from 2012 to 2021.

Annual national literature output was identified (Figure 2), in which the fastest growth occurred between 2019 and 2020. The United States of America (USA) (3512), Canada (909), Italy (898), China (824), and England (701) were the top 5 productive countries in terms of VTE anticoagulation research. The top 10 institutional contributors, productive funding agencies, research fields, and authors are presented in Figure S1. United States Department of Health and Human Services was the highest funding agency (596), followed by the National Institutes of Health (NIH), USA (561), Bayer Ag (331), National Heart Lung Blood Institute (298), and Pfizer (263) (Figure S1B). Cardiovascular system cardiology (3088), hematology (2600), general internal medicine (1631), surgery (1167), and pharmacology pharmacy (779) were the top 5 research domains (Figure S1C).

### 3.2. Analysis of Coauthorship

**Figure 3:**
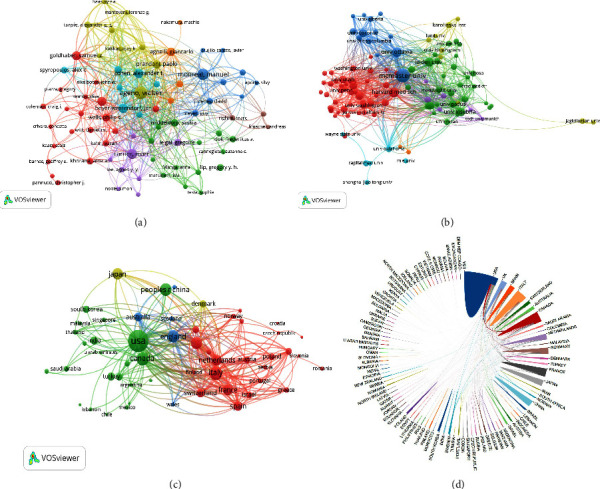
Bibliometric analysis of the coauthorship. (a) The cooperation of authors; (b) the cooperation of institutions; (c) and (d) the cooperation of countries or regions. Different colors indicate different clusters, the circle size indicates the number of publications, and the thickness of lines indicates the strength of the linkage.

Totally, 45595 authors have published papers focusing on VTE anticoagulation. We applied VOSviewer to analyze 77 authors who owned more than 20 publications (Figure 3(a)). Ageno W from the University of Insubria, who mainly focuses on surgical anticoagulation, has published 117 articles that have been cited 3873 times with a total link strength of 317. The main collaborators were Dentali F (link strength with Ageno W, 21) from the University of Insubria and Goldhaber SZ (link strength with Ageno W, 19) from Harvard Medical School. McMaster University has published 395 related articles with 35845 citations and a total link strength of 1222 (Figure 3(b)). The primary partner organizations are the Thrombosis and Atherosclerosis Research Institution (link strength with McMaster University, 83) and the University of Ottawa (link strength with McMaster University, 59).

The national cooperation network map (Figures 3(c) and 3(d)) shows that 61 out of 115 countries have published relevant articles over 10. The USA contributed the most documents, 3505, citing 89355 items and a total link strength of 2492. The closest cooperation nations with the USA were Canada, Germany, and England, with a link strength of 393, 236, and 226, respectively. The top ten coauthorship link strength countries, organizations, and authors in VTE anticoagulation topics are listed in Table S2.

### 3.3. Analysis of Citations

The top 10 most-cited documents in research on anticoagulation for venous thromboembolism (VTE) are presented in Table 1, while the top 100 cited papers are listed in Table S3. The majority of these articles were clinical guidelines that focused on prophylaxis and treatment of VTE. Additionally, articles that specifically addressed VTE anticoagulation in populations with cancer, surgery, antiphospholipid syndrome (APS), and pregnancy were also abundant. Clinical trials studying the efficacy and safety of nonvitamin K antagonist oral anticoagulants (NOACs) for anticoagulation in VTE, as well as research on the combination of thrombotic disease and COVID-19, have also garnered significant attention. The average number of citations for the top 100 most-cited papers was 545, with a range from 194 to 3104.

The ranking of the 10 most active journals, authors, organizations, and countries in publications on VTE anticoagulation is listed in Table 2. Chest, with an impact factor of 9.41, was the most-cited journal and produced 54 articles that received 15253 citations. Agnelli G from the University of Perugia, who contributed 59 pieces of literature, was the most-cited author (cited 8416 times) (Figure S2A). McMaster University was ranked the highest-cited institution, having published 395 articles that received 35845 citations (Figure S2B). In addition, the USA, the most-cited nation, had 3505 papers cited 89355 times, with a total link strength of 60415 (Figure S2C).

### 3.4. Analyses of Bibliographic Coupling and Cocitation

The results of bibliographic coupling analysis for authors, documents, journals, and countries are presented in Figure S3. In the network visualization of documents, 8 clusters were identified. The largest cluster, displayed in red, contained 46 items and focused on oral anticoagulants for the treatment of VTE. One representative article in this cluster was published in the New England Journal of Medicine in 2012 by Buller HR. The cocitation network maps for authors, references, and journals are indicated in Figure S4. Schulman S was the most cocited author, Kearon C, 2016, Chest, v149, p315, doi 10.1016/j.chest.2015.11.026 was the most cocited reference, and the New England Journal of Medicine was the representative journal.

### 3.5. Analyses of Keywords and Hotspots

**Figure 4:**
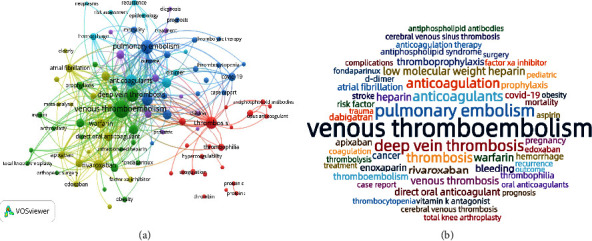
Bibliometric analysis of the keywords. (a): The cooccurrence of author keywords. Different colors indicate different clusters, the circle size indicates the number of occurrences, and the thickness of lines indicates the strength of the linkage. (b): The word cloud of keywords. The font size represents the frequency of occurrences.

The co-occurrence keyword analysis is presented in Figure 4(a). A total of 9803 author keywords were identified, and after removing duplicates, 94 met the threshold of 40 and finally enrolled in cooccurrence analysis. The most frequent keyword was “venous thromboembolism” (occurrences, 2142; total link strength, 4738), which had strong links to “pulmonary embolism” (occurrences, 1209; link strength, 430), “deep vein thrombosis” (occurrences, 1060; link strength, 391), “anticoagulants” (occurrences, 810; link strength, 273), “anticoagulation” (occurrences, 776; link strength, 246), and “rivaroxaban” (occurrences, 543; link strength, 189) (Figure 4(a)). A word cloud was also created to show the occurrence rates of keywords (Figure 4(b)).

**Figure 5:**
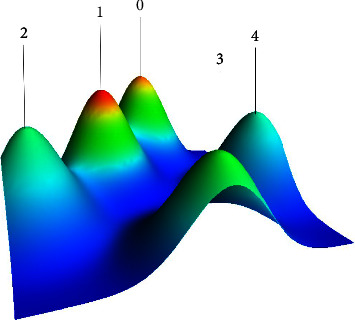
Mountain visualization map of high-frequency keywords biclustering in documents of VTE anticoagulant. Each hill represents a cluster, the distance between the mountains indicates the similarity between clusters, the height of the slope is proportional to the similarity of keywords within the cluster, the volume is proportional to the number of keywords contained in the cluster, and the color of the peak is proportional to the internal standard deviation of the cluster. Red means low standard deviation, while blue means high standard deviation.

**Figure 6:**
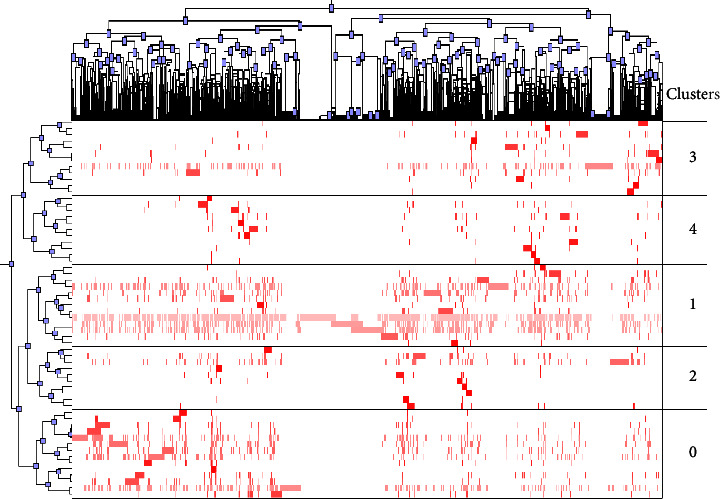
Matrix visualization map of high-frequency keywords biclustering in documents of VTE anticoagulant. Row labels are high-frequency keywords, and column labels are source documents. The left dendrogram represents the connection between high-frequency keywords, and the upper dendrogram represents the connection between source documents. Black horizontal lines separate clusters. Color represents the value in the original data matrix, and the deeper red color reveals a higher frequency of keywords.

A biclustering analysis of keywords was performed simultaneously to identify hotspots. Keywords were extracted from the retrieved articles, and synonyms were merged. High-frequency keywords, defined as those occurring over 50 times, are listed in Table S4. Figures 5 and 6 show the mountain and matrix visualization diagrams of high-frequency keywords-source articles matrix, with specific keywords of clusters as shown in Table S5. Five clusters were identified after adjusting the clustering parameter configuration as follows: (0): oral anticoagulant therapy for VTE; (1): prophylaxis for VTE patients combined with surgery and cancer; (2): parenteral anticoagulant treatment for VTE; (3): thrombosis characteristics in unique settings: children, people with APS and COVID-19; (4): clinical guidelines on the epidemiology, risk factors, diagnosis, treatment, and prognosis of VTE. The peak of clusters 0 and 1 was red and steep, indicating a relatively high concentration of similarity in research topics within these clusters. These clusters may reflect the hot topics in VTE anticoagulation research over the past decade.

### 3.6. Analyses of Themes and Trend Topics

**Figure 7:**
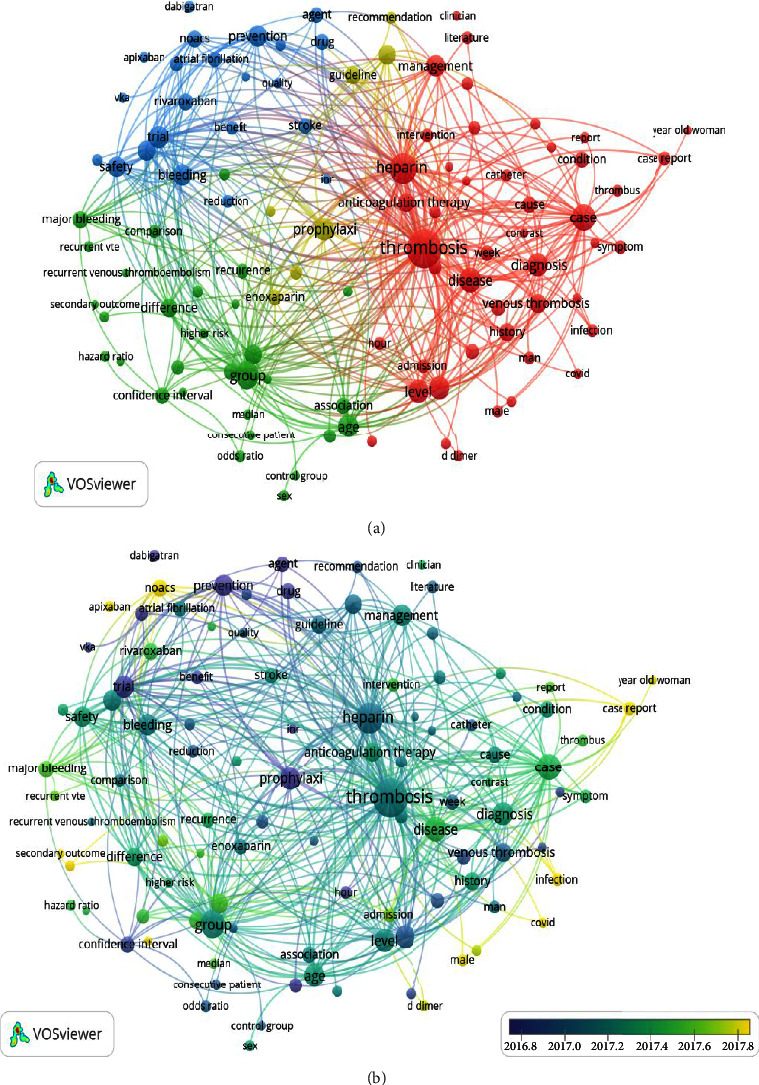
Bibliometric analysis of theme terms. (a): Distribution of the theme terms. (b): Overlay visualization map of the topic trends. The circle size indicates the number of occurrences, the thickness of the lines indicates the strength of linkage, and the different colors indicate different clusters (a) and the average publication year (b), respectively.

A total of 177 terms occurred more than 250 times.  Figure 7(a) shows four theme clusters of VTE anticoagulation in different colors. The red cluster involved comprehensive guidelines around VTE, including the causes, diagnosis, and treatment of VTE. The green cluster referred to the primary and secondary outcomes of VTE. The blue cluster focused on the efficacy and safety of oral anticoagulants for VTE. Also, the yellow cluster concerned parenteral anticoagulation prophylaxis of VTE. The overlay visualization map of the trend topics is demonstrated in Figure 7(b). As the year of publication approached, the color changed from yellow to purple. The result of overlay visualization of themes indicated that NOACs (occurrences, 993; average publication year, 2018.66) and COVID-19 (occurrences, 380; average publication year, 2020.74) were emerging trend topics.

## 4. Discussion

This study draws a comprehensive overview of the current research status and hotspots in the field of VTE anticoagulation based on an analysis of the 9692 documents. The number of articles in this field is growing rapidly, with the USA, Canada, and Italy being the top three productive countries. The keyword “Venous thromboembolism” had close links to “anticoagulants” and “rivaroxaban,” indicating that rivaroxaban, as a representative of NOACs, is a critical point in the treatment of VTE. The main focus of the articles included in the analysis was on clinical guidelines, special patient populations, NOACs application, and parenteral therapy of VTE anticoagulation. Future research interests will likely include evidence-based medicine for NOACs and studies on VTE patients combined with COVID-19.

### 4.1. Contributors in the VTE Anticoagulation Field

The rising annual number of publications on VTE anticoagulation suggests that this topic remains a hot topic. The largest publication volume is in the USA, which may be attributed to the abundant research workers and rich funding agencies [27]. Citations were a relatively proximate way to assess the impact of the published research [28]. Most of the highly cited countries, documents, and journals were located in North America and Europe, representing the high quality of articles in these areas. The top-cited author, Agnelli G, primarily concentrated on assessing oral factor Xa inhibitors for VTE and thromboprophylaxis for cancer-associated VTE patients, suggesting that these two aspects might be of current concern [29–33].

### 4.2. International Collaborations

International collaboration has been shown to bring various benefits to the development of medical discipline [34]. However, there was no information on international cooperation in the field of VTE anticoagulation. The USA was found to be the bellwether country in collaborating with other countries, while Canada and European countries also had strong links and produced a considerable amount of literature. However, countries such as China and Japan ranked as the top 4 and top 6, and most productive countries, respectively, had limited international collaboration impact outside the top 15. Therefore, cooperation between developed and developing countries or western and eastern countries should be further enhanced. The following methods may facilitate collaboration between nations, including importing foreign scholars, building an academic exchange platform, selecting experts and student visits, and increasing international academic collaboration funding [35–37].

### 4.3. Research Hotspots and Trends in the near Future

#### 4.3.1. Refine the Guidelines for Individualized Therapy

The management of VTE with anticoagulation was a complex process that needed comprehensive guidelines concerning epidemiology, risk factors, diagnosis, prevention, treatment, and prognosis of VTE to guide best practices. Therefore, high-quality guidelines such as evidence-based clinical practice guidelines of antithrombotic therapy and prevention of thrombosis, 9th edition formulated by the American College of Chest Physicians (ACCP-9), provided narrow therapeutic index anticoagulants as effective and safe as possible, and they have attracted significant attention in academic circles based on our analysis [38]. Meanwhile, the particular column of ACCP-9, including antithrombotic therapy for VTE [39], prevention of VTE in orthopedic surgery patients [40], prevention of VTE in nonorthopedic surgical patients [41], oral anticoagulant therapy [42], and prevention of VTE in nonsurgical patients, was also highly cited articles [43]. In addition, guidelines formulated by the European Society of Cardiology (ESC) [7], National Institute for Health and Care Excellence (NICE) [8], European Society for Vascular Surgery (ESVS) [44], and American Society of Hematology (ASH) [45] also recommend the best strategies for VTE anticoagulation. Still, they have fewer citations than the ACCP-9 guidelines, probably due to their relatively recent publication. Normative anticoagulation recommended by guidelines is not equal to effective anticoagulation. Individualized anticoagulation therapy should be adopted for different patients. The risk stratification of VTE adopting correlation risk scoring model and bleeding risk scoring model may be potential hotspots in future guidelines, which will be helpful for clinicians to screen patients with the best prevention benefits and then develop individualized prevention strategies.

#### 4.3.2. NOACs Application in VTE Anticoagulation

For decades, vitamin K antagonists (VKAs) and low molecular weight heparins (LMWHs) have been the primary anticoagulants used for managing VTE. Recently, the use of NOACs, particularly the direct factor Xa inhibitor rivaroxaban, has gained extensive attention due to its advantages, such as a single target mechanism, oral administration, no routine monitoring, and a lower risk of food-drug interaction [46]. The five III clinical trials, HOKUSAI-VTE [47], EINSTEIN-DVT [48], EINSTEIN-PE [30], AMPLIFY [31], RE-COVER [49], and RE-COVER II [50], have been highly cited, which were the robust evidence of NOACs. Also, the secondary analysis of these trials also occupied a high impact on this field [51]. Consequently, NOACs are recommended as the first-line treatment for general VTE patients and have rapidly become a research hotspot. In addition, the recommendation of NOACs in other clinical settings, especially cancer-associated VTE, was also undergoing extensive development [52–55]. Therefore, further research is needed to assess the risks or benefits of NOACs in different oncologic settings [56].

#### 4.3.3. VTE Anticoagulation in Special Situations

The thrombotic or bleeding risk of VTE in special populations, including orthopedic surgery, cancer, children, pregnant women, and APS, is higher than that in usual patients, posing certain challenges for decision-making clinicians [57–61]. In our analysis, we found that the leading research point in VTE patients undergoing orthopedic surgery is pharmacologic and mechanical thromboprophylaxis, in which LMWHs, fondaparinux, low-dose heparin, adjusted-dose VKAs, and aspirin are recommended anticoagulants in major orthopedic surgeries, and LMWHs has established its front-line treatment position [62, 63]. On the contrary, in patients with cancer-associated VTE, NOACs gradually become an alternative to LMWHs alone or LMWHs concurrently with a VKA [52]. The anticoagulation selection in different tumor types is diverse. For example, a high risk of venous thrombosis often accompanies active and advanced tumors. Therefore, the future topic is how to select anticoagulant drugs for this population while combining applicability and safety. Additionally, for pediatric and pregnant VTE, clinical options were based on traditional anticoagulant regimens, mainly belonging to off-label drugs and lacking good compliance. Anticoagulation of pregnant VTE will continue concerned with the impact of medication on pregnancy, fetus, and even lactation, as well as alternative treatments for heparin-induced thrombocytopenia. Dabigatran and rivaroxaban have been approved for the indications of pediatric VTE in 2021, which indicates the evaluation regarding the efficacy and safety of NOACs in the real world will be the future direction for those people [64]. Finally, in APS patients with low-risk VTE, NOACs are selected due to warfarin intolerance or previous unstable international normalized ratio of warfarin. Therefore, whether to continue NOACs would be a concerning issue for these people [65].

#### 4.3.4. Parenteral Anticoagulation in VTE Patients

Cluster 3 inferred that the studies concerning VTE parenteral anticoagulation were mainly focused on major orthopedic surgery, especially total hip arthroplasty (THA) and total knee arthroplasty (TKA). Heparin, LMWHs, and fondaparinux were high-frequency keywords. The ACCP-9 guidelines clearly recommend LMWHs over fondaparinux in patients with THA or TKA, regardless of the duration of surgery [66]. However, fondaparinux, relying on the low adverse reaction strength, has also become an alternative for heparin-induced thrombocytopenia patients. Meanwhile, the timing of parenteral anticoagulant therapy, preoperative, postoperative, or perioperative period, is the research direction for further refinement of clinical treatment decisions [67].

#### 4.3.5. VTE Patients Combined with COVID-19

The novel COVID-19 pandemic has swept the globe in recent three years. As a universal cardiovascular disease, VTE has emerged as an essential consideration for COVID-19 patients. The incidence of VTE in the COVID-19 population was relatively high, which might be attributed to COVID-19 leading to systemic coagulation activation and the potential drug-drug interactions between investigational therapies for COVID-19 and established agents used for VTE [68]. Meanwhile, hospitalized patients share similar risk factors for VTE, including advanced age, obesity, immobility, history of cancer, admission to intensive care units, and prior VTE history, with a higher risk of VTE [69, 70]. Besides the incidence, the diagnosis and treatment of VTE combined with COVID-19 patients also had extensive attention. However, the current medical knowledge of COVID-19 is still not comprehensive and in-depth. Therefore, the research frontiers in this field are devoted to conducting clinical trials to offer reliable and high-quality anticoagulation strategies [71].

### 4.4. Strengths and Limitations

Our study was the first bibliometric analysis focused on the articles involved in VTE anticoagulation. However, this study presents some limitations. First, although the WoSCC database was quite authoritative, other databases such as PubMed, Scopus, and Google Scholar were not considered in our analysis. Therefore, follow-up research should include a multidatabase to avoid high-quality research omission. Second, an inherent bias of bibliometric articles is that recently published studies lacked adequate citation time. Third, the phenomenon of self-cite may influence the accuracy of citation analysis. Fourth, the TS search, which included keywords plus, might cover several unrelated articles and reduce the accuracy of the included literature. Furthermore, some research hotspots may be lost due to the exclusion of reviews and non-English articles. These shortcomings need to be addressed and improved in future studies.

## 5. Conclusion

We conducted a bibliometric analysis based on the WOSCC database to study the characteristics of VTE anticoagulation research over the past decade. The research output is generally on the rise. Globally, the USA is the leading country in this field. The most influential documents, journals, and institutions were also from North America. Meanwhile, the cooperation and exchanges of countries and institutions need to strengthen, especially in developing countries or Asia. Agnelli G is an outstanding contributor to this domain. Currently, the research on VTE anticoagulation mainly focuses on the updation of VTE anticoagulation guidelines, assessment of the NOACs application, VTE management in special settings, and exploration of VTE combined with COVID-19, which may also be the trend of future research. The results of this analysis will facilitate researchers to identify cooperations, follow research hotspots, and predict the frontiers of VTE anticoagulation research.

## Figures and Tables

**Table 1:** The top ten most-cited articles in the field of VTE anticoagulation.

Rank	Title	Journal	Publication year	Citations
1	Guidelines for the early management of patients with acute ischemic stroke a guideline for healthcare professionals from the american heart association/American stroke association	Stroke	2013	3104
2	Antithrombotic therapy for VTE disease CHEST guideline and expert panel report	Chest	2016	2800
3	2014 ESC guidelines on the diagnosis and management of acute pulmonary embolism the task force for the diagnosis and management of acute pulmonary embolism of the european society of cardiology (ESC)	European Heart Journal	2014	2042
4	Antithrombotic therapy for VTE disease antithrombotic therapy and prevention of thrombosis, 9th ed: American college of chest physicians evidence-based clinical practice guidelines	Chest	2012	2003
5	Oral rivaroxaban for the treatment of symptomatic pulmonary embolism	New England Journal of Medicine	2012	1567
6	Oral apixaban for the treatment of acute venous thromboembolism	New England Journal of Medicine	2013	1397
7	High risk of thrombosis in patients with severe SARS-CoV-2 infection: a multicenter prospective cohort study	Intensive Care Medicine	2020	1321
8	Prevention of VTE in Orthopedic surgery patients antithrombotic therapy and prevention of thrombosis, 9th ed: American college of chest physicians evidence-based clinical practice guidelines	Chest	2012	1255
9	Prevention of VTE in nonorthopedic surgical patients antithrombotic therapy and prevention of thrombosis, 9th ed: American college of chest physicians evidence-based clinical practice guidelines	Chest	2012	1175
10	Edoxaban versus warfarin for the treatment of symptomatic venous thromboembolism	New England Journal of Medicine	2013	1155

**Table 2:** The top ten most active journals, authors, organizations, and countries in the field of VTE anticoagulation.

Journals	Cited times	No. of documents
Chest	15253	54
New England Journal of Medicine	14870	38
Journal of Thrombosis and Haemostasis	10397	302
Thrombosis Research	8137	565
Thrombosis and Haemostasis	7595	280
Blood	4487	61
Journal of Thrombosis and Thrombolysis	4467	294
Stroke	3547	23
Circulation	2860	29
Seminars in Thrombosis and Haemostasis	2290	140
Authors		
Agnelli G	8416	59
Bounameaux H	7552	32
Schulman S	7258	63
Kearon C	7168	25
Raskob GE	6672	33
Buller HR	6068	36
Crowther M	5723	32
Weitz JI	5707	49
Goldhaber SZ	5285	63
Prandoni P	5269	55
Organizations		
Mcmaster University	35845	395
University of Ottawa	13851	194
University of Amsterdam	11925	127
University of Washington	11919	104
Thrombosis and Atherosclerosis Research Institution	9035	84
University of Oklahoma	8383	72
University of Perugia	7841	93
Mcgill University	7502	88
Harvard University	7470	88
Thrombosis Research Institution	7127	45
Countries		
USA	89355	3505
Canada	52486	906
Italy	30904	896
England	29795	698
Netherlands	28968	564
Germany	27056	697
France	24172	562
Spain	16037	416
Switzerland	16004	274
Australia	12543	314

## Data Availability

The data supporting the findings of this study are available in the article.

## Supplementary Materials

Supplementary Materials Table S1: Retrieval strategy in Web of Science Core Collection database; Figure S1: The top 10 institutional contributors, productive funding agencies, research fields, and authors of Venous Thromboembolism (VTE) anticoagulation articles exported from Web of Science core collection; Table S2: Top ten coauthorship link strength countries, organizations, and authors; Table S3: The top 100 most-cited articles in VTE anticoagulation; Figure S2: Bibliometric analysis of the citation; Figure S3: Bibliometric analysis of the bibliographic coupling; Figure S4: Bibliometric analysis of the cocitation; Table S4: Highly frequency keywords from the included documents on VTE anticoagulation; Table S5: The specific keywords of clusters in the mountain diagram and matrix diagram.
