# Supplementary material for: Global Research Hotspots in Venous Thromboembolism Anticoagulation: A Knowledge-Map Analysis from 2012 to 2021
Source: J Interv Cardiol. 2023 Nov 17;2023:4717271. doi: 10.1155/2023/4717271 (PMC10673674; doi:10.1155/2023/4717271)
Supplement: Supplementary Materials — Table S1: Retrieval strategy in Web of Science Core Collection database; Figure S1: The top 10 institutional contributors, productive funding agencies, research fields, and authors of Venous Thromboembolism (VTE) anticoagulation articles exported from Web of Science core collection; Table S2: Top ten coauthorship link strength countries, organizations, and authors; Table S3: The top 100 most-cited articles in VTE anticoagulation; Figure S2: Bibliometric analysis of the citation; Figure S3: Bibliometric analysis of the bibliographic coupling; Figure S4: Bibliometric analysis of the cocitation; Table S4: Highly frequency keywords from the included documents on VTE anticoagulation; Table S5: The specific keywords of clusters in the mountain diagram and matrix diagram. [file 4717271.f1.docx]

**Table S1.** Retrieval strategy in Web of Science Core Collection database

| **#1** | #1 TS= 'pulmonary embolism' OR 'venous thromboembolism' OR 'VTE' OR 'DVT' OR 'PTE' OR 'PE' OR 'deep vein thrombosis' OR 'venous thrombosis' |
| --- | --- |
| **#2** | #2 TS= 'anticoagulant' OR 'oral anticoagulant' OR 'anticoagulant therapy' OR 'phenindione' OR 'heparin' OR 'enoxaparin' OR 'dalteparin' OR 'tinzaparin' OR 'danaparoid' OR 'fondaparinux' OR 'bivalirudin' OR 'low molecular weight heparin' OR 'LMWH' OR 'new oral anticoagulants' OR 'novel oral anticoagulants' OR 'non-Vitamin K antagonist' OR 'direct oral anticoagulants' OR 'oral thrombin inhibitors' OR 'oral factor Xa inhibitors' OR 'oral factor Xa inhibitors' OR 'NOACs' OR 'DOACs' OR 'DTI' OR 'DTIs' OR 'dabigatran' OR 'apixaban' OR 'rivaroxaban' OR 'edoxaban' OR 'argatroban' OR 'argatroban' OR 'non-Vitamin K antagonists' OR 'VKA' OR 'VKAs' OR 'vitamin K antagonist' OR 'warfarin' OR 'acenocoumarol' OR 'phenprocoumon' |
| **#3** | #1 AND #2 |

**
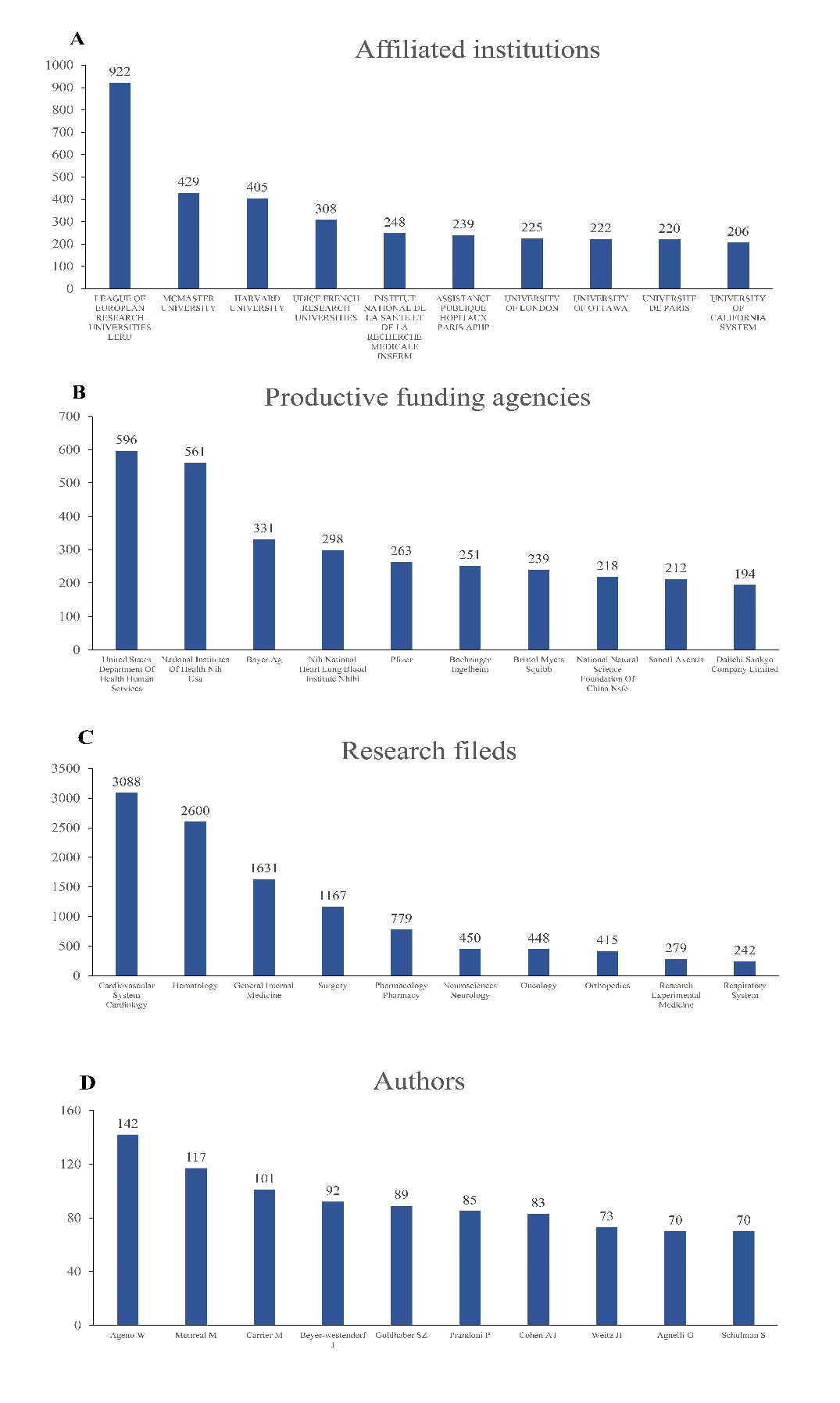

Figure S1.** Bibliometric analysis of Venous Thromboembolism (VTE) anticoagulation articles exported from Web of Science core collection. A: Top ten institutional contributors; B: Top ten productive funding agencies; C: Top ten active research fields; D: Top ten published authors.

**Table S2.** Top ten co-authorship link strength countries, organizations, and authors

| Country | Documents | Citations | Total link strength |
| --- | --- | --- | --- |
| USA | 3505 | 89355 | 2492 |
| Canada | 906 | 52486 | 1816 |
| Germany | 697 | 27056 | 1741 |
| Italy | 896 | 30904 | 1721 |
| England | 698 | 29795 | 1610 |
| Netherlands | 564 | 28968 | 1385 |
| France | 562 | 24172 | 1205 |
| Spain | 416 | 16037 | 1008 |
| Switzerland | 274 | 16004 | 832 |
| Belgium | 148 | 8905 | 607 |
| Organization | Documents | Citations | Total link strength |
| Mcmaster University | 395 | 35845 | 1222 |
| University of Ottawa | 194 | 13851 | 637 |
| Harvard Medical School | 187 | 5109 | 489 |
| Thrombosis & Atherosclerosis Research Institution | 84 | 9035 | 420 |
| University of Insubria | 137 | 7076 | 396 |
| University of Washington | 104 | 11919 | 352 |
| University of Amsterdam | 127 | 11925 | 339 |
| University College London | 69 | 5782 | 306 |
| Duke University | 115 | 5858 | 293 |
| University of Oklahoma | 72 | 8383 | 285 |
| Author | Documents | Citations | Total link strength |
| Ageno W | 117 | 3873 | 317 |
| Prandoni P | 55 | 5269 | 247 |
| Bounameaux H | 32 | 7552 | 194 |
| Goldhaber SZ | 63 | 5285 | 188 |
| Beyer-Westendorf J | 71 | 2158 | 187 |
| Cohen AT | 69 | 1670 | 180 |
| Weitz JI | 49 | 5707 | 174 |
| Wells PS | 51 | 3690 | 166 |
| Verhamme P | 35 | 4808 | 162 |
| Carrier M | 79 | 3533 | 160 |

**Table S3.** The top 100 most-cited articles in VTE anticoagulation

| Rank | Title | Journal | Publication year | Citations |
| --- | --- | --- | --- | --- |
| 1 | Guidelines for the Early Management of Patients With Acute Ischemic Stroke A Guideline for Healthcare Professionals From the American Heart Association/American Stroke Association | Stroke | 2013 | 3104 |
| 2 | Antithrombotic Therapy for VTE Disease CHEST Guideline and Expert Panel Report | Chest | 2016 | 2800 |
| 3 | 2014 ESC Guidelines on the Diagnosis and Management of Acute Pulmonary Embolism The Task Force for the Diagnosis and Management of Acute Pulmonary Embolism of the European Society of Cardiology (ESC) | European Heart Journal | 2014 | 2042 |
| 4 | Antithrombotic Therapy for VTE Disease Antithrombotic Therapy and Prevention of Thrombosis, 9th ed: American College of Chest Physicians Evidence-Based Clinical Practice Guidelines | Chest | 2012 | 2003 |
| 5 | Oral Rivaroxaban for the Treatment of Symptomatic Pulmonary Embolism | New England Journal of Medicine | 2012 | 1567 |
| 6 | Oral Apixaban for the Treatment of Acute Venous Thromboembolism | New England Journal of Medicine | 2013 | 1397 |
| 7 | High risk of thrombosis in patients with severe SARS-CoV-2 infection: a multicenter prospective cohort study | Intensive Care Medicine | 2020 | 1321 |
| 8 | Prevention of VTE in Orthopedic Surgery Patients Antithrombotic Therapy and Prevention of Thrombosis, 9th ed: American College of Chest Physicians Evidence-Based Clinical Practice Guidelines | Chest | 2012 | 1255 |
| 9 | Prevention of VTE in Nonorthopedic Surgical Patients Antithrombotic Therapy and Prevention of Thrombosis, 9th ed: American College of Chest Physicians Evidence-Based Clinical Practice Guidelines | Chest | 2012 | 1175 |
| 10 | Edoxaban versus Warfarin for the Treatment of Symptomatic Venous Thromboembolism | New England Journal of Medicine | 2013 | 1155 |
| 11 | Oral Anticoagulant Therapy Antithrombotic Therapy and Prevention of Thrombosis, 9th ed: American College of Chest Physicians Evidence-Based Clinical Practice Guidelines | Chest | 2012 | 881 |
| 12 | Prevention of VTE in Nonsurgical Patients Antithrombotic Therapy and Prevention of Thrombosis, 9th ed: American College of Chest Physicians Evidence-Based Clinical Practice Guidelines | Chest | 2012 | 860 |
| 13 | Thrombotic Thrombocytopenia after ChAdOx1 nCov-19 Vaccination | New England Journal of Medicine | 2021 | 839 |
| 14 | Apixaban for Extended Treatment of Venous Thromboembolism | New England Journal of Medicine | 2013 | 818 |
| 15 | Perioperative Management of Antithrombotic Therapy Antithrombotic Therapy and Prevention of Thrombosis, 9th ed: American College of Chest Physicians Evidence-Based Clinical Practice Guidelines | Chest | 2012 | 816 |
| 16 | 2019 ESC Guidelines for the diagnosis and management of acute pulmonary embolism development Toed in collaboration with the European Respiratory Society (ERS) | European Heart Journal | 2020 | 794 |
| 17 | Evidence-Based Management of Anticoagulant Therapy Antithrombotic Therapy and Prevention of Thrombosis, 9th ed: American College of Chest Physicians Evidence-Based Clinical Practice Guidelines | Chest | 2012 | 792 |
| 18 | Antithrombotic Therapy in Neonates and Children Antithrombotic Therapy and Prevention of Thrombosis, 9th ed: American College of Chest Physicians Evidence-Based Clinical Practice Guidelines | Chest | 2012 | 784 |
| 19 | Incidence of Venous Thromboembolism in　Hospitalized Patients with COVID-19 | Journal of Thrombosis and Haemostasis | 2020 | 766 |
| 20 | Fibrinolysis for Patients with Intermediate-Risk Pulmonary Embolism | New England Journal of Medicine | 2014 | 761 |
| 21 | Edoxaban for the Treatment of Cancer-Associated Venous Thromboembolism | New England Journal of Medicine | 2018 | 741 |
| 22 | Extended Use of Dabigatran, Warfarin, or Placebo in Venous Thromboembolism | New England Journal of Medicine | 2013 | 672 |
| 23 | 2018 ESC Guidelines for the Management of Cardiovascular Diseases During Pregnancy | European Heart Journal | 2018 | 615 |
| 24 | Treatment of Acute Venous Thromboembolism With Dabigatran or Warfarin and Pooled Analysis | Circulation | 2014 | 614 |
| 25 | Thrombosis and Thrombocytopenia after ChAdOx1 nCoV-19 Vaccination | New England Journal of Medicine | 2021 | 585 |
| 26 | A Randomized Trial of Genotype-Guided Dosing of Warfarin | New England Journal of Medicine | 2013 | 582 |
| 27 | Venous Thromboembolism Prophylaxis and Treatment in Patients With Cancer: American Society of Clinical Oncology Clinical Practice Guideline Update | Journal of Clinical Oncology | 2013 | 580 |
| 28 | Long-term Outcome after Additional Catheter-directed Thrombolysis Versus Standard Treatment for Acute Iliofemoral Deep Vein Thrombosis: a Randomised Controlled Trial | Lancet | 2012 | 575 |
| 29 | VTE, Thrombophilia, Antithrombotic Therapy, and Pregnancy Antithrombotic Therapy and Prevention of Thrombosis, 9th ed: American College of Chest Physicians Evidence-Based Clinical Practice Guidelines | Chest | 2012 | 571 |
| 30 | COVID-19 and Coagulation: Bleeding and Thrombotic Manifestations of SARS-CoV-2 Infection | Blood | 2020 | 564 |
| 31 | Parenteral Anticoagulants Antithrombotic Therapy and Prevention of Thrombosis, 9th ed: American College of Chest Physicians Evidence-Based Clinical Practice Guidelines | Chest | 2012 | 553 |
| 32 | Pulmonary Embolism and Deep Vein Thrombosis | Lancet | 2012 | 546 |
| 33 | Treatment and Prevention of Heparin-Induced Thrombocytopenia Antithrombotic Therapy and Prevention of Thrombosis, 9th ed: American College of Chest Physicians Evidence-Based Clinical Practice Guidelines | Chest | 2012 | 523 |
| 34 | Comparison of an Oral Factor Xa Inhibitor With Low Molecular Weight Heparin in Patients With Cancer With Venous Thromboembolism: Results of a Randomized Trial (SELECT-D) | Journal of Clinical Oncology | 2018 | 491 |
| 35 | Direct Oral Anticoagulants Compared with Vtamin K Antagonists for Acute Venous Thromboembolism: Evidence from Phase 3 Trials | Blood | 2014 | 483 |
| 36 | The Procoagulant Pattern of Patients with COVID-19 Acute Respiratory Distress Syndrome | Journal of Thrombosis and Haemostasis | 2020 | 477 |
| 37 | Venous Thromboembolism Prophylaxis and Treatment in Patients With Cancer: American Society of Clinical Oncology Clinical Practice Guideline Update 2014 | Journal of Clinical Oncology | 2015 | 476 |
| 38 | Long-Term Outcomes of Patent Foramen Ovale Closure or Medical Therapy after Stroke | New England Journal of Medicine | 2017 | 468 |
| 39 | Age-Adjusted D-Dimer Cutoff Levels to Rule Out Pulmonary Embolism The ADJUST-PE Study | Jama-Journal of the American Medical Association | 2014 | 457 |
| 40 | The Epidemiology of Venous Thromboembolism | Journal of Thrombosis and Thrombolysis | 2016 | 454 |
| 41 | Aspirin for Preventing the Recurrence of Venous Thromboembolism | New England Journal of Medicine | 2012 | 450 |
| 42 | Venous Thromboembolism Prophylaxis and Treatment in Patients With Cancer: ASCO Clinical Practice Guideline Update | Journal of Clinical Oncology | 2020 | 433 |
| 43 | Pathologic Antibodies to Platelet Factor 4 after ChAdOx1 nCoV-19 Vaccination | New England Journal of Medicine | 2021 | 414 |
| 44 | Thrombolysis for Pulmonary Embolism and Risk of All-Cause Mortality, Major Bleeding, and Intracranial Hemorrhage A Meta-analysis | Jama-Journal of the American Medical Association | 2014 | 412 |
| 45 | Effect of Mon-specific Reversal Agents on Anticoagulant Activity of Dabigatran and Rivaroxaban | Thrombosis and Haemostasis | 2012 | 408 |
| 46 | Tinzaparin vs Warfarin for Treatment of Acute Venous Thromboembolism in Patients With Active Cancer A Randomized Clinical Trial | Jama-Journal of the American Medical Association | 2015 | 399 |
| 47 | Pulmonary Arterial Thrombosis in COVID-19 With Fatal Outcome Results From a Prospective, Single-Center, Clinicopathologic Case Series | Annals of Internal Medicine | 2020 | 395 |
| 48 | Rivaroxaban for Thromboprophylaxis in Acutely Ill Medical Patients | New England Journal of Medicine | 2013 | 394 |
| 49 | Antithrombotic and Thrombolytic Therapy for Valvular Disease Antithrombotic Therapy and Prevention of Thrombosis, 9th ed: American College of Chest Physicians Evidence-Based Clinical Practice Guidelines | Chest | 2012 | 385 |
| 50 | National Trends in Ambulatory Oral Anticoagulant Use | American Journal of Medicine | 2015 | 376 |
| 51 | Low-Dose Aspirin for Preventing Recurrent Venous Thromboembolism | New England Journal of Medicine | 2012 | 371 |
| 52 | Semuloparin for Thromboprophylaxis in Patients Receiving Chemotherapy for Cancer | New England Journal of Medicine | 2012 | 371 |
| 53 | Scientific and Standardization Committee Communication: Clinical Guidance on the Diagnosis, Prevention, and Treatment of Venous Thromboembolism in Hospitalized Patients with COVID-19 | Journal of Thrombosis and Haemostasis | 2020 | 367 |
| 54 | Rivaroxaban or Aspirin for Extended Treatment of Venous Thromboembolism | New England Journal of Medicine | 2017 | 365 |
| 55 | Efficacy and Safety of Dabigatran Etexilate and Warfarin in "Real-World" Patients With Atrial Fibrillation A Prospective Nationwide Cohort Study | Journal of the American College of Cardiology | 2013 | 350 |
| 56 | Apixaban to Prevent Venous Thromboembolism in Patients with Cancer | New England Journal of Medicine | 2019 | 350 |
| 57 | 2019 ESC Guidelines for the Diagnosis and Management of Acute Pulmonary Embolism Developed in Collaboration with the European Respiratory Society (ERS) | European Respiratory Journal | 2019 | 336 |
| 58 | Factor XI Antisense Oligonucleotide for Prevention of Venous Thrombosis | New England Journal of Medicine | 2015 | 336 |
| 59 | Prevention, Diagnosis, and Treatment of VTE in Patients With Coronavirus Disease 2019 CHEST Guideline and Expert Panel Report | Chest | 2020 | 334 |
| 60 | Effectiveness and Safety of Novel Oral Anticoagulants as Compared with Vitamin K Antagonists in the Treatment of Acute Symptomatic Venous Thromboembolism: A Systematic Review and Meta- analysis | Journal of Thrombosis and Haemostasis | 2014 | 330 |
| 61 | Pharmacomechanical Catheter-Directed Thrombolysis for Deep-Vein Thrombosis | New England Journal of Medicine | 2017 | 325 |
| 62 | Moderate Pulmonary Embolism Treated With Thrombolysis (from the "MOPETT" Trial) | American Journal of Cardiology | 2013 | 320 |
| 63 | Rivaroxaban vs Warfarin in High-risk Patients with Antiphospholipid Syndrome | Blood | 2018 | 311 |
| 64 | Rates, Management, and Outcome of Rivaroxaban Bleeding in Daily Care: Results From the Dresden NOAC Registry | Blood | 2014 | 308 |
| 65 | Comparative Effectiveness and Safety of Non-Vitamin K Antagonist Oral Anticoagulants and Warfarin in Patients with Atrial Fibrillation: Propensity Weighted Nationwide Cohort Study | Bmj-British Medical Journal | 2016 | 307 |
| 66 | Regional Anesthesia in the Patient Receiving Antithrombotic or Thrombolytic Therapy: American Society of Regional Anesthesia and Pain Medicine Evidence-Based Guidelines (Fourth Edition) | Regional Anesthesia and Pain Medicine | 2018 | 302 |
| 67 | The Postthrombotic Syndrome: Evidence-Based Prevention, Diagnosis, and Treatment Strategies A Scientific Statement From the American Heart Association | Circulation | 2014 | 297 |
| 68 | Extended Thromboprophylaxis with Betrixaban in Acutely Ill Medical Patients | New England Journal of Medicine | 2016 | 290 |
| 69 | Antithrombotic and Thrombolytic Therapy for Ischemic Stroke Antithrombotic Therapy and Prevention of Thrombosis, 9th ed: American College of Chest Physicians Evidence-Based Clinical Practice Guidelines | Chest | 2012 | 289 |
| 70 | Myocardial Ischemic Events in Patients with Atrial Fibrillation Treated With Dabigatran or Warfarin in the RE-LY (Randomized Evaluation of Long-Term Anticoagulation Therapy) Trial | Circulation | 2012 | 288 |
| 71 | Predicting Disease Recurrence in Patients with Previous Unprovoked Venous Thromboembolism: A Proposed Prediction Score (DASH) | Journal of Thrombosis and Haemostasis | 2012 | 286 |
| 72 | Anticoagulant Treatment is Associated with Decreased Mortality in Severe Coronavirus Disease 2019 Patients With Coagulopathy | Journal of Thrombosis and Haemostasis | 2020 | 285 |
| 73 | Rivaroxaban for Thromboprophylaxis in High-Risk Ambulatory Patients with Cancer | New England Journal of Medicine | 2019 | 284 |
| 74 | Apixaban for the Treatment of Venous Thromboembolism Associated with Cancer | New England Journal of Medicine | 2020 | 276 |
| 75 | American Society of Hematology 2018 Guidelines for Management of Venous Thromboembolism: Prophylaxis for Hospitalized and Nonhospitalized Medical Patients | Blood Advances | 2018 | 275 |
| 76 | Impact of Dabigatran on a Large Panel of Routine or Specific Coagulation Assays Laboratory Recommendations for Monitoring of Dabigatran Etexilate | Thrombosis and Haemostasis | 2012 | 271 |
| 77 | Effect of a Retrievable Inferior Vena Cava Filter Plus Anticoagulation vs Anticoagulation Alone on Risk of Recurrent Pulmonary Embolism A Randomized Clinical Trial | Jama-Journal of the American Medical Association | 2015 | 268 |
| 78 | Biomarkers and Surrogate Endpoints in Clinical Trials | Statistics in Medicine | 2012 | 264 |
| 79 | Risk Factors and Basic Mechanisms of Chronic Thromboembolic Pulmonary Hypertension: a Current Understanding | European Respiratory Journal | 2013 | 252 |
| 80 | Guidance on the Emergent Reversal of Oral Thrombin and Factor Xa Inhibitors | American Journal of Hematology | 2012 | 250 |
| 81 | Thromboembolism and Anticoagulant Therapy During the COVID-19 Pandemic: Interim Clinical Guidance from the Anticoagulation Forum | Journal of Thrombosis and Thrombolysis | 2020 | 249 |
| 82 | Early Thrombus Removal Strategies for Acute Deep Venous Thrombosis: Clinical Practice Guidelines of the Society for Vascular Surgery and the American Venous Forum | Journal of Vascular Surgery | 2012 | 245 |
| 83 | Rivaroxaban Versus Warfarin to Treat Patients with Thrombotic Antiphospholipid Syndrome, With or Without Systemic Lupus Erythematosus (RAPS): A Randomised, Controlled, Open-Label, Phase 2/3, Non-Inferiority Trial | Lancet Haematology | 2016 | 225 |
| 84 | Systemic Thrombolytic Therapy for Acute Pulmonary Embolism: A Systematic Review and Meta-Analysis | European Heart Journal | 2015 | 225 |
| 85 | American Society of Hematology 2018 Guidelines for Management of Venous Thromboembolism: Heparin-Induced Thrombocytopenia | Blood Advances | 2018 | 222 |
| 86 | In Vivo Roles of Factor XII | Blood | 2012 | 215 |
| 87 | Gemcitabine Versus Gemcitabine Plus Dalteparin Thromboprophylaxis in Pancreatic Cancer | European Journal of Cancer | 2012 | 213 |
| 88 | HALO 202: Randomized Phase II Study of PEGPH20 Plus Nab- Paclitaxel/Gemcitabine Versus Nab- Paclitaxel/Gemcitabine in Patients With Untreated, Metastatic Pancreatic Ductal Adenocarcinoma | Journal of Clinical Oncology | 2018 | 213 |
| 89 | Management and Outcomes of Major Bleeding During Treatment With Dabigatran or Warfarin | Circulation | 2013 | 212 |
| 90 | The Impact of Bleeding Complications in Patients Receiving Target-Specific Oral Anticoagulants: a Systematic Review and Meta-Analysis | Blood | 2014 | 209 |
| 91 | US Case Reports of Cerebral Venous Sinus Thrombosis With Thrombocytopenia After Ad26.COV2.S Vaccination, March 2 to April 21, 2021 | Jama-Journal of the American Medical Association | 2021 | 208 |
| 92 | Efficacy and Safety of Anticoagulation on Patients With Cirrhosis and Portal Vein Thrombosis | Clinical Gastroenterology and Hepatology | 2012 | 207 |
| 93 | COVID-19 Coagulopathy in Caucasian Patients | British Journal of Haematology | 2020 | 205 |
| 94 | A Factor XIIa Inhibitory Antibody Provides Thromboprotection in Extracorporeal Circulation Without Increasing Bleeding Risk | Science Translational Medicine | 2014 | 204 |
| 95 | Evaluation of the Anti-Factor Xa Chromogenic Assay for the Measurement of  Rivaroxaban Plasma Concentrations Using Calibrators and Controls | Thrombosis and Haemostasis | 2012 | 203 |
| 96 | Aspirin or Enoxaparin Thromboprophylaxis for Patients with Newly Diagnosed Multiple Myeloma Treated with Lenalidomide | Blood | 2012 | 197 |
| 97 | Guidance for the Practical Management of the Direct Oral Anticoagulants (DOACs) in VTE Treatment | Journal of Thrombosis and Thrombolysis | 2016 | 196 |
| 98 | Safety, Pharmacokinetics and Pharmacodynamics of Multiple Oral Doses of Apixaban, a Factor Xa Inhibitor, in Healthy Subjects | British Journal of Clinical Pharmacology | 2013 | 196 |
| 99 | 2017 ACC Expert Consensus Decision Pathway on Management of Bleeding in Patients on Oral Anticoagulants | Journal of the American College of Cardiology | 2017 | 195 |
| 100 | Post-thrombotic Syndrome after Catheter-Directed Thrombolysis for Deep Vein Thrombosis (Cavent): 5-Year Follow-Up Results of an Open-Label, Randomised Controlled Trial | Lancet Haematology | 2016 | 194 |

**
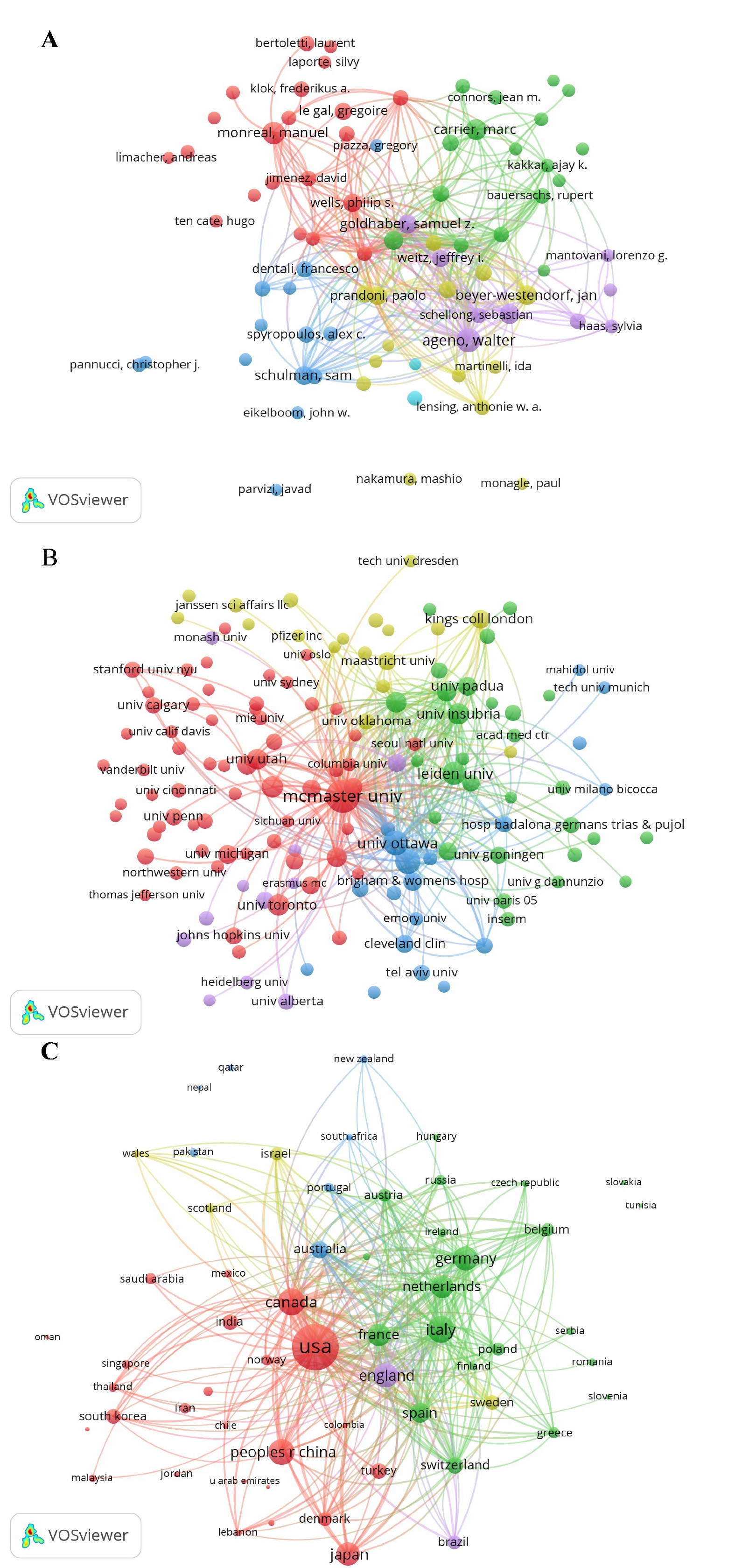
**

**Figure S2.** Bibliometric analysis of the citation. A: The citation of authors; B: the citation of institutions; C: the citation of countries or regions. Different colors indicate different clusters, the circle size indicates the counts of citations, and the thickness of lines indicates the strength of linkage.


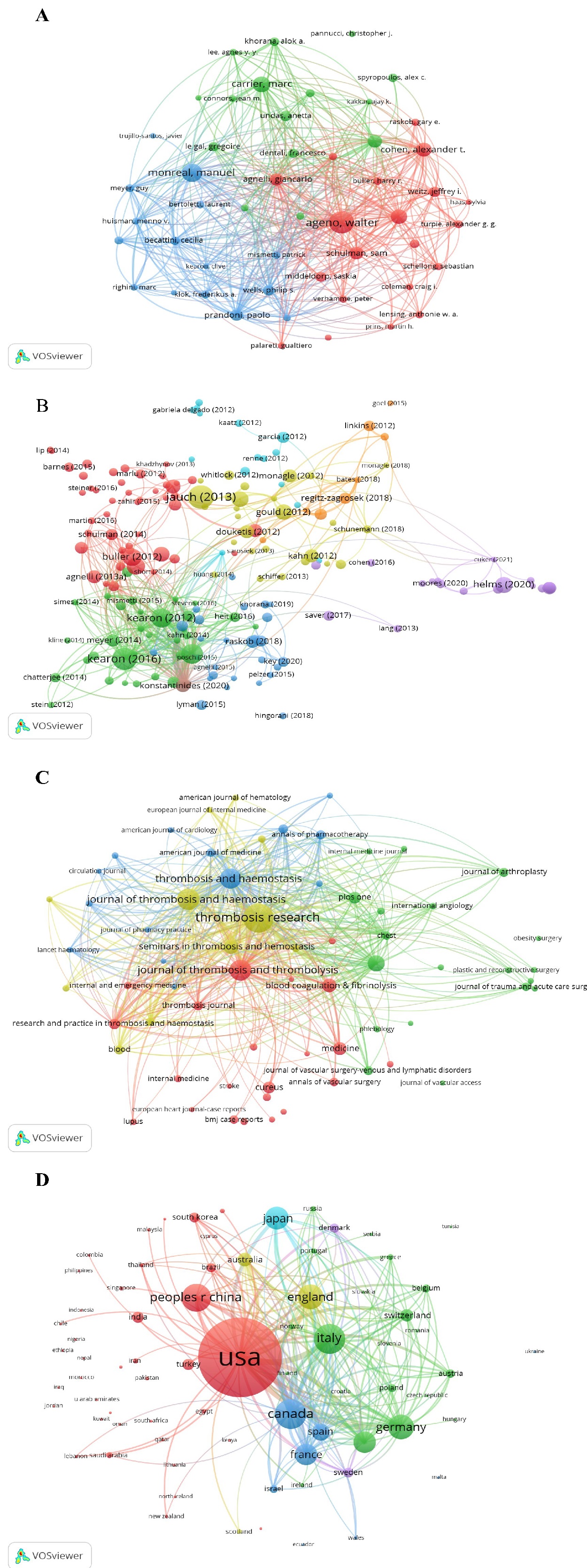


**Figure S3.** Bibliometric analysis of the bibliographic coupling. A: Bibliographic coupling of authors; B: bibliographic coupling of documents; C: bibliographic coupling of journals; D: bibliographic coupling of countries or regions. Different colors indicate different clusters, the circle size indicates the counts of citations, and the thickness of lines indicates the strength of linkage.


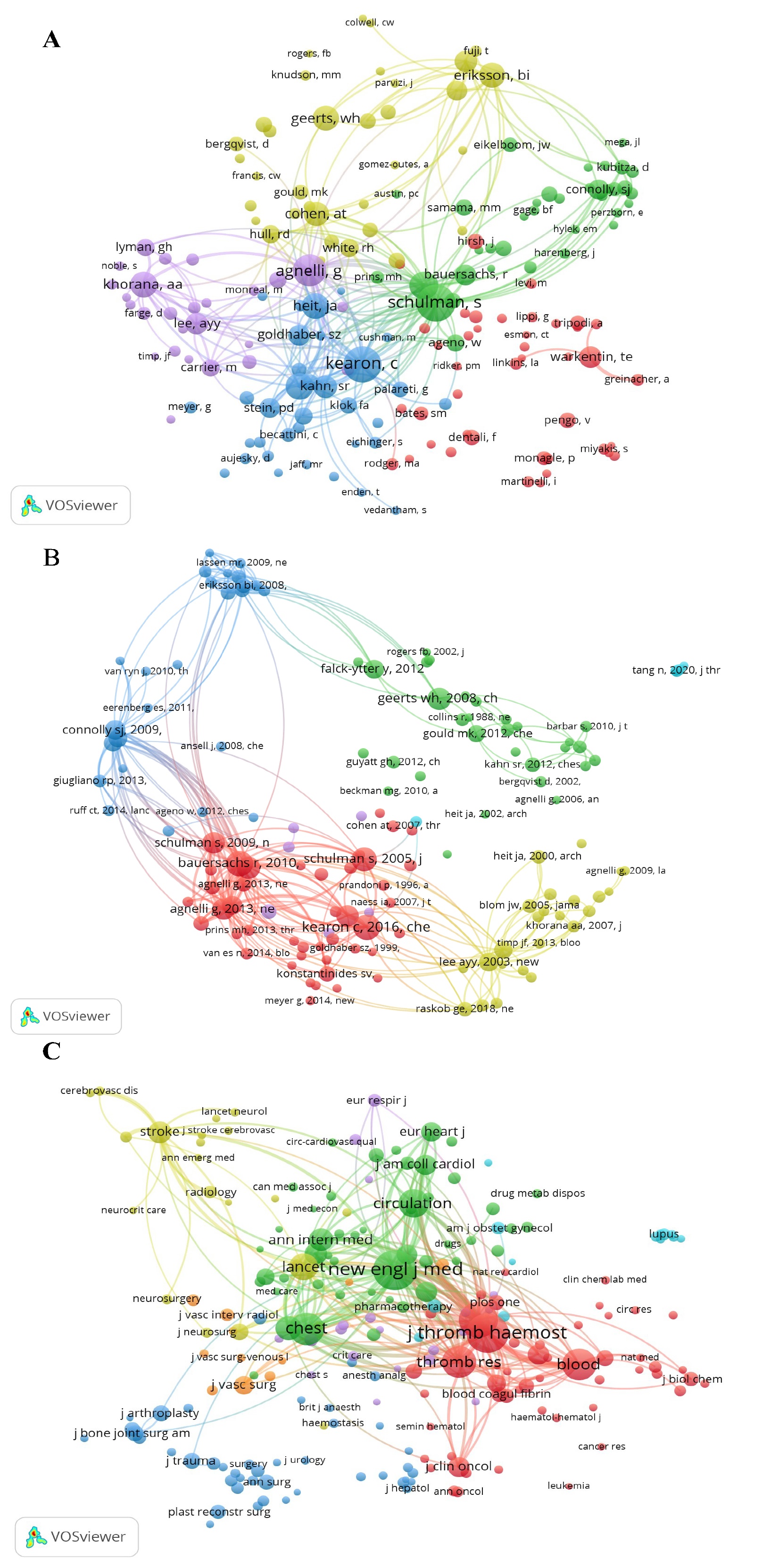


**Figure S4.** Bibliometric analysis of the co-citation. A: Co-citation of authors; B: co-citation of references; C: co-citation of sources. Different colors indicate different clusters, the circle size indicates the counts of citations, and the thickness of lines indicates the strength of linkage.

**Table S4.** Highly-frequency keywords from the included documents on VTE anticoagulation

| Rank | Keywords | Occurrence | Percentage (%) | Cumulative percentage (%) |
| --- | --- | --- | --- | --- |
| 1 | VTE | 2549 | 6.6484 | 6.6484 |
| 2 | PE | 1191 | 3.1064 | 9.7548 |
| 3 | DVT | 1067 | 2.7830 | 12.5378 |
| 4 | anticoagulant | 809 | 2.1101 | 14.6479 |
| 5 | thrombosis | 779 | 2.0318 | 16.6797 |
| 6 | anticoagulation | 775 | 2.0214 | 18.7011 |
| 7 | rivaroxaban | 543 | 1.4163 | 20.1174 |
| 8 | NOACs | 536 | 1.3980 | 21.5154 |
| 9 | LMWH | 508 | 1.3250 | 22.8404 |
| 10 | warfarin | 461 | 1.2024 | 24.0428 |
| 11 | heparin | 401 | 1.0459 | 25.0887 |
| 12 | bleeding | 350 | 0.9129 | 26.0016 |
| 13 | thromboprophylaxis | 332 | 0.8659 | 26.8675 |
| 14 | cancer | 304 | 0.7929 | 27.6604 |
| 15 | COVID-19 | 284 | 0.7407 | 28.4011 |
| 16 | enoxaparin | 271 | 0.7068 | 29.1080 |
| 17 | AF | 264 | 0.6886 | 29.7966 |
| 18 | thromboembolism | 253 | 0.6599 | 30.4564 |
| 19 | prophylaxis | 251 | 0.6547 | 31.1111 |
| 20 | apixaban | 220 | 0.5738 | 31.6849 |
| 21 | dabigatran | 216 | 0.5634 | 32.2483 |
| 22 | hemorrhage | 196 | 0.5112 | 32.7595 |
| 23 | cerebral venous thrombosis | 190 | 0.4956 | 33.2551 |
| 24 | pregnancy | 182 | 0.4747 | 33.7298 |
| 25 | thrombophilia | 176 | 0.4591 | 34.1888 |
| 26 | stroke | 175 | 0.4564 | 34.6453 |
| 27 | risk factors | 162 | 0.4225 | 35.0678 |
| 28 | antiphospholipid syndrome | 152 | 0.3965 | 35.4643 |
| 29 | mortality | 143 | 0.3730 | 35.8372 |
| 30 | D-dimer | 124 | 0.3234 | 36.1607 |
| 31 | edoxaban | 120 | 0.3130 | 36.4737 |
| 32 | recurrence | 120 | 0.3130 | 36.7866 |
| 33 | coagulation | 116 | 0.3026 | 37.0892 |
| 34 | VKAs | 108 | 0.2817 | 37.3709 |
| 35 | thrombolysis | 104 | 0.2713 | 37.6421 |
| 36 | aspirin | 103 | 0.2686 | 37.9108 |
| 37 | anticoagulant therapy | 100 | 0.2608 | 38.1716 |
| 38 | case report | 97 | 0.2530 | 38.4246 |
| 39 | fondaparinux | 86 | 0.2243 | 38.6489 |
| 40 | surgery | 85 | 0.2217 | 38.8706 |
| 41 | obesity | 84 | 0.2191 | 39.0897 |
| 42 | thrombocytopenia | 80 | 0.2087 | 39.2984 |
| 43 | factor Xa inhibitor | 77 | 0.2008 | 39.4992 |
| 44 | prognosis | 76 | 0.1982 | 39.6974 |
| 45 | trauma | 76 | 0.1982 | 39.8957 |
| 46 | VTE prophylaxis | 73 | 0.1904 | 40.0861 |
| 47 | treatment | 73 | 0.1904 | 40.2765 |
| 48 | antiphospholipid antibodies | 73 | 0.1904 | 40.4669 |
| 49 | children | 71 | 0.1852 | 40.6521 |
| 50 | heparin-induced thrombocytopenia | 70 | 0.1826 | 40.8346 |
| 51 | epidemiology | 68 | 0.1774 | 41.0120 |
| 52 | meta analysis | 66 | 0.1721 | 41.1841 |
| 53 | prevention | 65 | 0.1695 | 41.3537 |
| 54 | total hip arthroplasty | 61 | 0.1591 | 41.5128 |
| 55 | oral anticoagulants | 56 | 0.1461 | 41.6588 |
| 56 | complications | 55 | 0.1435 | 41.8023 |
| 57 | protein C | 52 | 0.1356 | 41.9379 |
| 58 | diagnosis | 52 | 0.1356 | 42.0736 |
| 59 | elderly | 51 | 0.1330 | 42.2066 |
| 60 | intracranial hemorrhage | 48 | 0.1252 | 42.3318 |

**Table S5.** The specific keywords of clusters in the mountain diagram and matrix diagram

| 0 | factor Xa inhibitor |
| --- | --- |
|  | edoxaban |
|  | dabigatran |
|  | rivaroxaban |
|  | warfarin |
|  | bleeding |
|  | NOACs |
|  | VKAs |
|  | oral anticoagulants |
|  | stroke |
|  | AF |
|  | anticoagulant |
|  | hemorrhage |
| 1 | prevention |
|  | pregnancy |
|  | thromboembolism |
|  | anticoagulant |
|  | LMWH |
|  | cancer |
|  | surgery |
|  | prophylaxis |
|  | VTE |
|  | PE |
|  | DVT |
|  | thromboprophylaxis |
|  | trauma |
| 2 | obesity |
|  | enoxaparin |
|  | heparin |
|  | meta analysis |
|  | aspirin |
|  | VTE prophylaxis |
|  | complications |
|  | intracranial hemorrhage |
|  | total hip arthroplasty |
|  | fondaparinux |
| 3 | coagulation |
|  | protein C |
|  | thrombophilia |
|  | children |
|  | cerebral venous thrombosis |
|  | antiphospholipid syndrome |
|  | antiphospholipid antibodies |
|  | thrombosis |
|  | COVID-19 |
|  | case report |
|  | heparin-induced thrombocytopenia |
|  | thrombocytopenia |
| 4 | elderly |
|  | D-dimer |
|  | recurrence |
|  | risk factors |
|  | epidemiology |
|  | mortality |
|  | prognosis |
|  | anticoagulant therapy |
|  | thrombolysis |
|  | diagnosis |
|  | treatment |
